# Editorial: Reviews in cellular neuropathology

**DOI:** 10.3389/fncel.2026.1821476

**Published:** 2026-04-13

**Authors:** Alessandro Tozzi, Chao Deng, Dirk M. Hermann

**Affiliations:** 1 Section of Physiology and Biochemistry, Department of Medicine and Surgery, University of Perugia, Perugia, Italy; 2 School of Medical, Indigenous and Health Sciences, and Molecular Horizons, University of Wollongong, Wollongong, NSW, Australia; 3 Department of Neurology, University Hospital Essen, University of Duisburg-Essen, Essen, Germany

**Keywords:** blood-brain barrier, epigenetic, ferroptosis, multiomics, neurodegenerative diseases, neuroinflammation, neurosteroids, synaptic transmission

Exciting advances are currently made in the translational neuroscience field, enabling better understandings of cellular responses to brain injuries via single cell transcriptomics, proteomics and metabolomics, connecting findings in different research fields by big data set analysis and artificial intelligence tools, and providing frameworks for the treatment of clinical conditions, such as Alzheimer's disease, for which until recently no causatively acting therapeutics interventional existed. In this rapidly advancing field, the Research Topic “*Reviews in cellular neuropathology*” brings together 11 contributions that collectively delineate a genuine frontier in translational neuroscience. As editors of this Topic, we indeed observe a clear shift toward approaches that move beyond traditional disciplinary boundaries, embracing the intrinsic complexity of central nervous system (CNS) disorders through integrative and multidisciplinary perspectives. This evolution reflects the growing awareness that neurological diseases cannot be fully understood by isolating single pathways, but rather require a systems-level view that connects molecular and cellular events to physiological processes, such as synaptic dysfunction and network failure.

The relevance of this field lies in its ability to bridge finely regulated cellular mechanisms with higher-order dysfunction and therapeutic strategies. This conceptual framework is particularly critical for several neurodegenerative conditions, in which decades of research have yet to yield effective disease-modifying treatments. A recurring theme emerging from contemporary neuroscience is the loss of homeostatic balance: when molecular control mechanisms governing ion channel activity, gene regulation, protein stability, or immune responses are disrupted, the CNS progressively shifts toward maladaptive plasticity and neurodegeneration. Understanding how these alterations converge at the synaptic and circuit levels is therefore essential for translating basic discoveries into meaningful clinical advances. Within this context, the articles included in this Research Topic provide examples how diverse molecular players, experimental models, conceptual frameworks and computational methods can be integrated to clarify disease mechanisms and identify new therapeutic opportunities.

A recurring message across contemporary studies, highlighted throughout this Research Topic, is the importance of integrating diverse experimental models and diseases to accelerate discovery and translation. For example, Ding et al. in this Research Topic showed that memory processes and neuronal development can be influenced by the transmembrane protein DPP6, which acts as a critical modulator of the potassium channel (Kv4.2) function, with its dysregulation implicated not only in Alzheimer's disease but also in motor disorders such as amyotrophic lateral sclerosis.

To achieve progress enabling clinical human application, bridging research across species has significant importance. In this context, Yadav et al. illustrated how simple organisms such as the nematode *Caenorhabditis elegans* provide powerful platforms for rapidly interrogating complex genetic mutations and for screening candidate pharmacological interventions. Accordingly, they showed how this model can be effective in studying a group of neglected genetic disorders, namely GNAO1 encephalopathies, primarily occurring due to *de novo* mutations in the Gαo protein-encoding gene.

To enable clinical translatability, previously established approaches are increasingly complemented by more advanced inducible cell type-specific murine models and human clinical investigations. In this context, Kawato et al. demonstrated that locally synthesized neuro-estrogens and neuro-androgens play an essential role in hippocampus-dependent cognition and synaptic plasticity, challenging the traditional view that cognitive regulation depends exclusively on circulating endocrine signals.

The translational relevance of basic research becomes particularly evident when novel mechanisms of neural damage are identified. Huang et al. reported that ferroptosis, an iron-dependent form of regulated cell death, represents a key contributor to CNS injuries such as stroke and traumatic brain injury, opening new therapeutic avenues based on iron chelation and redox modulation strategies. At the same time, Moskal and Michalak highlighted the dual role of tight junction proteins at the blood-brain barrier, which not only protect neural tissue from edema in glial tumors but also represent potential targets for controlled modulation to enhance chemotherapeutic drug delivery. Similarly, Zhao et al. reframed heatstroke as a disorder primarily driven by dysregulation of warm-sensitive neurons rather than a purely thermal insult, implicating neuroinflammatory cascades that extend along the gut-liver-brain axis.

As reported by Kocakusak et al., growing attention has been directed toward tRNA-derived fragments, small molecules playing essential roles in maintaining cellular homeostasis, in disease pathogenesis. Interestingly, these fragments also function as epigenetic and post-translational regulators rather than passive degradation products, positioning them as promising biomarkers and potential therapeutic targets for neurological and psychiatric conditions, including Parkinson's disease and epilepsy.

This epigenetic complexity is further reviewed by Xu et al., which present the innovative concept of the retina as an extension of the CNS, detailing the changes in DNA methylation patterns in diseases such as diabetic retinopathy, age-related macular degeneration, and glaucoma. The exploration is further extended from the retina to the brain, where DNA methylation not only guides neuronal development but also serves as a molecular “clock” and a diagnostic window for disorders such as schizophrenia and autism.

Together with pharmacological and molecular advances, contemporary neuroscience increasingly recognizes the contribution of lifestyle factors and holistic approaches to brain health. Canonichesi et al. showed that physical exercise improves sleep quality in synucleinopathies such as Parkinson's disease, thereby facilitating the clearance of toxic α-synuclein aggregates. In parallel, Silva et al. reported that environmental enrichment, through sensory and cognitive stimulation, can modulate autophagy, the brain's intrinsic cellular maintenance system, under adverse conditions. Complementing these findings, Yu et al. highlighted how compounds derived from traditional Chinese medicine may regulate microglia-driven neuroinflammation, promoting phenotypic shifts toward neuroprotective states that support neuronal repair and functional recovery in Alzheimer's disease.

Taken together, these converging lines of evidence convey a clear message: the future of neuroscience and medicine lies in the effective translation of fundamental discoveries, ranging from protein interactions and RNA fragments to regulated cell death pathways, into targeted and integrated therapeutic strategies. The convergence of basic molecular research, experimental models, advanced imaging techniques, novel delivery methods, pharmacological and non-pharmacological interventions represents the most promising path forward for addressing neurological and psychiatric disorders that pose an increasing global burden, reaffirming that scientific research is not an abstract attempt but a critical driver of meaningful improvements in patients' quality of life.

